# Financing national scale energy projects in developing countries – An economy-wide evaluation of Ghana's Bui Dam

**DOI:** 10.1016/j.eneco.2022.106065

**Published:** 2022-07

**Authors:** Victor Nechifor, Mohammed Basheer, Alvaro Calzadilla, Emmanuel Obuobie, Julien J. Harou

**Affiliations:** a European Commission Joint Research Centre, Seville, Spain; b Institute for Sustainable Resources, University College London, London, United Kingdom; c School of Mechanical, Aerospace and Civil Engineering, The University of Manchester, Manchester, United Kingdom; d Water Research Institute, Council for Scientific and Industrial Research, Accra, Ghana; e Civil, Geomatic and Environmental Engineering, University College London, London, United Kingdom

**Keywords:** Energy infrastructure development, Project finance, Computable general equilibrium, Developing countries, Water balance model, Hydrological uncertainty

## Abstract

Large energy infrastructure can imply special financing arrangements between governments in developing economies and investors or lenders. These arrangements can lead to economy-wide and sector-specific impacts which need to be considered in the project economic evaluation. By considering the case of the Bui Dam in Ghana, we use a macroeconomic approach to determine how the economic performance of critical energy infrastructure manifests during the construction, financing and operation phases. The analysis uses an integrated modelling framework that combines a Computable General Equilibrium (CGE) model of Ghana with a water balance model of the Lower Volta River Basin. The results highlight the importance of including indirect and induced effects, in addition to the direct effects from project operation, as they influence the scale and temporal evolution of the economic impacts. The collateral from the infrastructure loan agreement consisting in cocoa exports to China nearly doubles the project's positive GDP impact and has a significant multiplier effect over urban and rural household income compared to a standard commercial loan. We finish with a discussion of how the proposed investment-oriented modelling framework can contribute to ex-ante strategic assessments of proposed energy infrastructure in developing countries.

## Highlights

• The construction, financing and operation of a national energy project in Ghana is evaluated.
• The economy is simulated through an investment-oriented CGE model.
• Stochastic hydropower generation is estimated using a bio-physical system model.
• The inclusion of the construction and financing phases increases the project GDP value.
• Loan conditions with collateral are as important as the hydropower generation value.

## 1 Introduction

Large-scale infrastructure projects can have substantial economic, social and environmental implications (WCD, 2000; Bhattacharya et al., 2015; Ascensão et al., 2018; Thacker et al., 2019) imposing economy-wide impacts on GDP and employment that vary across project implementation and operation. For developing countries, there is evidence that infrastructure investment impacts employment and household income on a similar scale to foreign aid (Bhatia et al., 2008).

One essential facet to realizing large-scale projects in developing countries is access to finance. Infrastructure projects can be “large, lumpy, and infrequent; they often take more than one budget cycle to complete” Briceño-Garmendia et al. (2009). Without external financing and by relying only on internal public and private savings, the implementation of large-scale projects in developing countries may take a long time and can ‘crowd-out’ investment in other areas of the economy. At the same time, foreign lending or investment in developing countries with low credit rating can be accompanied by specific conditions that can generate their own economic implications, e.g., procurement and engineering contracts, trade arrangements with lending governments or fiscal incentives for private investors (Markkanen and Plummer Braeckman, 2019).

Economic development and structural change in national economies depend on the size of stocks and flows of social, human, natural, financial and manufactured capital (Maack and Davidsdottir, 2015). In low- and medium-income countries, investments in infrastructure can be in the order of one or two digits of the annual GDP (Briceño-Garmendia et al., 2009). Moreover, the achievement of global decarbonization targets will require considerable investments in clean power generation, building efficiency, electrification of the transport sector, and low carbon technologies in other sectors of the economy. The IPCC estimates that average annual investment in low-carbon energy technologies and energy efficiency would need to increase by a factor of six by 2050 compared to 2015 to limit the global temperature increase to 1.5 °C (Rogelj et al., 2018). Therefore, there is a use-case for an analytical framework that helps to assess the economy-wide impacts of such investments and their lending conditions. The proposed approach is relevant for the global South where two-thirds of the world's infrastructure investment is expected to occur in the next decades (NCE, 2016). Furthermore, Sub-Saharan Africa is a region where half of the infrastructure is financed through foreign lending and investment (Foster and Briceño-Garmendia, 2009) and where non-traditional lenders, e.g. the Chinese government, have a growing stake (Bräutigam and Gallagher, 2014; Gutman et al., 2015; IEA, 2016).

Several studies explored the economy-wide impacts of large-scale infrastructure in the area of energy, trade and agriculture in developing regions (Robinson et al., 2008; Strzepek et al., 2008; Ginting et al., 2015; Kahsay et al., 2015a; Ma et al., 2015a; Boehlert et al., 2017; Kahsay et al., 2019). Typically, they focus on identifying benefits from the operation (use) of the asset and put less or no attention on the impacts of project financing and construction. The emphasis in these studies is placed on assessing the direct impacts (e.g. trade costs reduction, electricity generation, irrigation water supply) more than the indirect or induced effects (e.g. supply chain impacts, income effects). The short-term impacts of the construction phase (i.e., economic activity and job creation) and the long-term implications of financing (i.e., source of funding, interest rates, payback periods, and other associated conditions) are omitted in these studies. However, they are key determinants of the economic case of large infrastructure such as dams (Bhatia et al., 2008). Estimating these effects goes beyond the standard evaluation of projects that use rates of return (WCD, 2000). For nuclear energy projects in the US, Oxford Economics (2008) found that the total economic effects of investment in new nuclear capacity are over three times larger than the direct effects when both indirect and induced effects are included.

For national-scale dam projects, previous studies used capital requirements as a criterion for dam portfolio development (Block and Strzepek, 2010; Jeuland and Whittington, 2014; Sridharan et al., 2019). To the best of our knowledge, Ma et al. (2015a) and Kahsay et al. (2015a) are the only studies to date that consider investment requirements for dam development in an economy-wide analysis framework. In Ma et al. (2015b) the financial requirements of dam construction were implicitly deducted from household savings. Kahsay et al. (2015b) introduced the financial needs of dam construction through an exogenous increase in domestic savings. This private financing of investments may have different effects depending on the macro-economic structure or closure rule. In addition, in both studies, the dam construction inputs (e.g. labour, engineering services) were not introduced as project-specific. Therefore the analysis did not reflect project cost structure. Furthermore, they used single-year static Computable General Equilibrium (CGE) models so the impacts on household consumption and government spending were not carried forward through the project lifetime.

This study introduces a dynamic single-country investment-oriented CGE model. The model includes a project-specific activity that captures the materials, capital, and labour requirements during the construction phase of the infrastructure - a large dam in this study. Project finance is included by considering four government-backed financing options (grants, bonds, public spending and resource-secured foreign loans). Economic impacts of infrastructure use are included through the addition of revenue streams from infrastructure services.

The model is used to estimate the economy-wide impacts of the Bui Dam, a multi-year storage dam in Ghana which started operating in 2013. The Bui Dam is an interesting case for study for project finance in developing countries because its investment requirements have been partly secured through a loan from the Chinese Government conditioned by a 20-year cocoa export agreement. We assess the economy-wide impacts of the Bui Dam across three phases: construction, financing, and operation. The direct impacts in terms of change in electricity generation are estimated using a bio-physical water balance model of the Lower Volta Basin that is calibrated based on satellite-based reservoir water level data. The water balance model accounts for both the additional electricity from the Bui Dam and the change in electricity generation from the Akosombo Dam located downstream from the Bui Dam.

The rest of the article is structured as follows: Section 2 describes the main characteristics of the proposed integrated CGE and bio-physical system simulation framework; Section 3 describes the applications of the models to Ghana and the Bui Dam; Section 4 discusses results, implications and limitations; Section 5 concludes.

## 2 Methods

We propose an analysis framework whereby a CGE model (InvestCGE) is linked to a spatially explicit, bio-physical system model. The economy model determines the macro-economic, sectoral and household-level impacts of large-scale infrastructure construction, financing and operation. The bio-physical system model determines the aggregate changes to infrastructure service levels in terms of hydropower generation considering hydrological uncertainty. The changes to hydropower supply are then transmitted to the CGE model as economic shocks to hydropower output.

### 2.1 Investment-oriented CGE model

#### 2.1.1 General characteristics

This study introduces the InvestCGE model, a single-country CGE model that assess the economy-wide impacts of investment and operation of infrastructure projects. InvestCGE is tailored to reflect direct, indirect and induced impacts of infrastructure investment across infrastructure project construction, financing, and operation. Large-scale projects have impacts that occur over long times; thus, InvestCGE was specified as a dynamic-recursive model with labour and capital supply being updated to follow population dynamics and capital formation, respectively. InvestCGE was developed based on the International Food Policy Research Institute (IFPRI) standard CGE model (Lofgren et al., 2002) and thus inherited its main characteristics that are suitable for economy-wide analyses in developing economies. These characteristics include (1) disaggregation of households by location (e.g. urban and rural) and labour by skill levels (e.g. skilled and unskilled workers), (2) inclusion of home (non-marketed) and subsistence consumption, and (3) the small open-economy assumption in which international trade is subject to duties and the national economy does not influence global commodity prices. Similar to other CGE models, InvestCGE assumes utility-maximizing households, cost-minimizing firms, and supply and demand parity across all commodity and factor markets. International trade is included through commodity flows between the national economy and the rest of the world, following the Armington assumption (Armington, 1969). This assumption implies that imports and domestic products are imperfect substitutes. Exports are governed by a Constant Elasticity of Transformation (CET) function through which domestic production adapts to foreign markets.

**Fig. 1:**
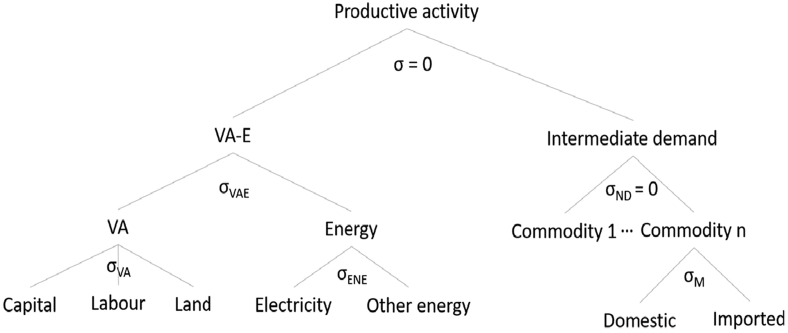
Production specification using nested Constant Elasticity of Substitution (CES) functions. Note: σ = elasticity of substitution; VA = value-added; VA-E = value-added-energy.

Production activities are specified through nested Constant Elasticity of Substitution (CES) functions. Fig. 1 shows the production structure adopted herein. To simulate the role of energy in the economy, the initial specification of production in the standard IFPRI CGE model was extended to include direct substitution possibilities between value-added and energy, similar to energy-oriented CGE models, e.g., Burniaux and Truong (2002) and Chateau et al. (2014). The value-added-energy (VA-E) bundle is then combined at the top level with the intermediate demand aggregate assuming perfect complementarity.

We modified the standard IFPRI model by introducing an upward slopping labour supply curve. Accordingly, the supply of labour was endogenised such that it increases with wages through a supply elasticity. InvestCGE also distinguishes between agricultural capital and capital used in industry, non-hydro energy, and services. Investment allocation across these two capital types is performed based on their relative rates of return.

#### 2.1.2 Characterization of infrastructure investment

InvestCGE includes modelling capabilities specific to the implementation of government-backed infrastructure projects These features are introduced across the construction, financing, and operation phases of the project as follows:

• Construction: project construction is added as an independent temporary economic activity occurring only during the construction phase. The construction activity uses labour, commodities, and services as inputs, reflecting the real costs of the dam. The model enables specification of directly imported machinery and parts that cannot be sourced domestically (e.g. hydropower turbines).
• Financing: single or combined financing sources can apply. Four financing options are implemented, namely government bonds, foreign loans, foreign grants and government spending. For government bonds and foreign loans, repayments to each financing source are calculated annually based on specified interest rates. Grants can be obtained by the government in the form of external aid which does not need to be paid back. Governments can decide to fully or partly finance an infrastructure project through their own means by deducting investment expenses from general public spending.
• Operation: project-specific rents attributed to the project capital are separated from other rents, i.e., capital, labour, and other operation and maintenance costs. This separation enables quantifying direct value-added resulting from project operation but also the resulting government revenue streams from the project.

**Fig. 2:**
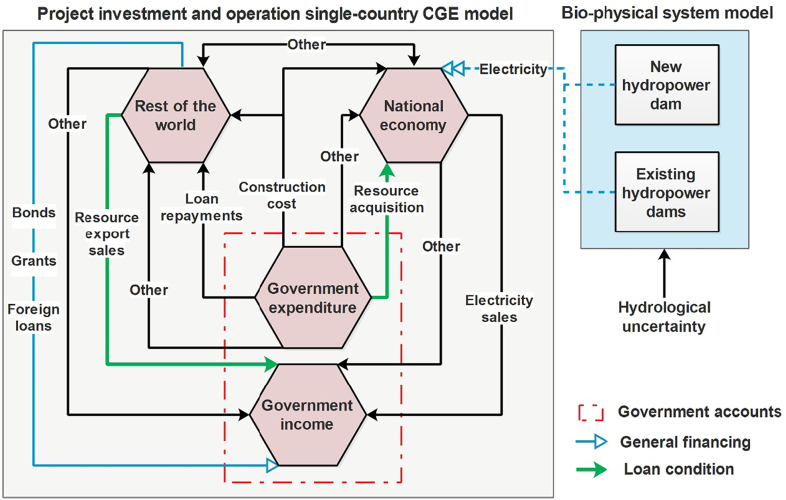
Monetary flows between the government accounts and the other accounts in InvestCGE. Note: for clarity, some accounts and transactions are aggregated in the figure.

Since InvestCGE focuses on the implementation of projects that are financed through governmental arrangements, the government income and expenditure accounts are central. Fig. 2 outlines the transactions between the government accounts and the other accounts during the three project implementation phases. Note that some accounts and transactions are aggregated in the figure for clarity. The two government accounts are impacted differently at each stage of project implementation. In the construction phase, project costs are included in the government budgetary expenditure. To match this spending, through the finance component of the model, the government can contract grants and loans, issue bonds or reduce expenditure on public services. Loans are repaid with interest for a specified period and can include grace periods. Preferential interest rates can be considered through resource export agreements with the lending entities (resource-secured loans), notably in developing regions where access to finance is difficult (Markkanen and Plummer Braeckman, 2019). The government would need to acquire those resources from the domestic market (at the domestic market price) and make them available for export under the negotiated conditions (e.g. guaranteed exported volumes at an agreed price). To consider these government spending and revenue impacts distinctly, the loan repayments and the resource export sales are considered as separate transactions in the model. Finally, once the project enters its operational phase, the government receives rents from the project capital utilization. In the case of hydropower dams, capital rents result from electricity sales from which operation and maintenance costs are deducted.

The macro closure rules of the model are based on the assumption of savings-driven investment. Therefore, total investment is variable and is based on household and firm propensity to save. This closure choice prevents steep reductions in the consumption of households due to temporary expansion in investment that results from the project implementation. The total spending of the government on both public services and loan repayments is set as a fixed share of total absorption, while government savings are flexible to clear the government account balance. To isolate the effect of project finance and loan repayment on the exchange rate, foreign savings are set to be exogenous. Therefore, the exchange rate in the model is variable.

### 2.2 Bio-physical system model

This section describes the mathematical formulation of the bio-physical system model. The application of the model to the Volta Basin is described in Section 3.3. As Fig. 2 shows, a bio-physical model was connected to InvestCGE to estimate hydropower generation and the rents that the government is expected to receive from the utilization of project capital. The bio-physical model is a rule-based water balance model that has a monthly time step. The model simulates reservoir storage, evaporation, and power generation in addition to channel flow and losses. The outflows from dams are simulated based on Eq. (1).

(1) $$O_{d,t}=I_{d,t}-{\mathrm{EV}}_{d,t}+\left(S_{d,t-1}-S_{d,t}\right)\forall d,t$$

where *t* is the time step (months), *d* is a set of dams (Bui and Akosombo), *O*_*d*, *t*_ is the outflow volume from the dam *d* at the time step *t*, *I*_*d*, *t*_ is the inflow to the reservoir of the dam *d* at the time step *t, EV*_*d*, *t*_ is the evaporation volume from the reservoir of the dam *d* at the time step *t*, *S*_*d*, *t*−1_ is the storage in the reservoir of the dam *d* at the time step *t-1*, and *S*_*d*, *t*_ is the storage in the reservoir of the dam *d* at the time step *t*. Reservoir evaporation (*EV*_*d*, *t*_) was calculated using periodic monthly evaporation rates (Eqs. (2), (3)):

(2) $${\mathrm{EV}}_{d,t}=A_{d,t}\times F_{d,t}\forall d,t$$

(3) $$A_{d,t}=f\left(S_{d,t-1}\right)\forall d,t$$

where *A*_*d*, *t*_ is the surface area of the reservoir of the dam *d* at the time step *t*, *F*_*d*, *t*_ is the reservoir evaporation rate in the month *t*.

Two types of dam operating rules are implemented to drive the model simulations: target power and target storage. The target power rule determines the reservoir storage that achieves a predefined power generation target while maintaining the reservoir between the dead storage and the maximum storage (Eqs. (4), (5)). The target storage rule assigns a predefined value to the reservoir storage and estimates power generation accordingly (Eqs. (5), (6), (7)).

(4) $$S_{d,t}=S_{d,t-1}-\left[\frac{P_{d}}{\gamma \times \left(H_{d,t}-T_{d}\right)\times \alpha_{d}}\right]\times t-{\mathrm{EV}}_{d,t}\;{\mathrm{MS}}_{d}\leq S_{d,t}\leq {\mathrm{XS}}_{d}$$

(5) $$H_{d,t}=f\left(S_{d,t-1}\right)\forall d,t$$

(6) $$S_{d,t}={\mathrm{ST}}_{d,t}\;{\mathrm{MS}}_{d}\leq S_{d,t}\leq {\mathrm{XS}}_{d}$$

(7) $$P_{d}=\gamma \times O_{d,t}\times \left(H_{d,t-1}-T_{d}\right)\times \alpha_{d}\forall d,t$$

where *P*_*d*_ is a constant power target for the dam *d*, *γ* is the specific weight of water, *H*_*d*, *t*_ is the water level of the reservoir of the dam *d* at the time step *t*, *T*_*d*_ is the tailwater level of the dam *d*, *α*_*d*_ is the efficiency of the turbines of the dam *d*, *MS*_*d*_ is the dead storage of the reservoir of the dam d, *XS*_*d*_ is the maximum storage of the reservoir of the dam d, and *ST*_*d*, *t*_ is the target storage of the reservoir of the dam *d* at the time step *t*.

Constant loss fractions were used to estimate channel losses according to Eq. (8). The channel loss volumes were deducted from the channel flows (Eq. (9)):

(8) $${\mathrm{CL}}_{c,t}=p_{c}\times I_{c,t}\forall c,t$$

(9) $$O_{c,t}=I_{c,t}-{\mathrm{CL}}_{c,t}\forall c,t$$

where *c* is the set of channels in the Volta Basin (Black Volta, White Volta, and Oti River), *CL*_*c*, *t*_ the loss volume from the channel *c* at the time step *t*, *p*_*c*_ is the loss fraction from the channel *c*, *I*_*c*, *t*_ is the inflow to the channel *c* at the time step *t*, *O*_*c*, *t*_ is the outflow from the channel c at the time step *t*.

## 3 Case study

### 3.1 General features

**Fig. 3:**
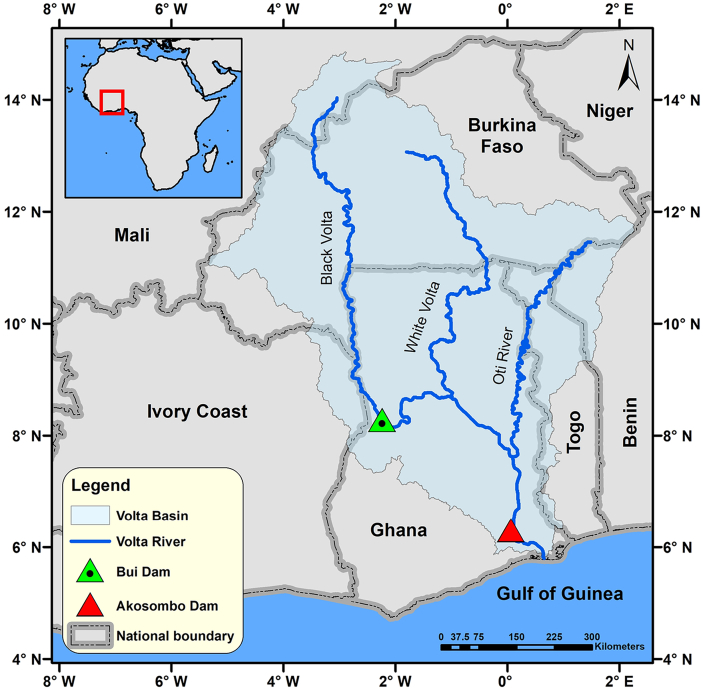
Main features of the study area.

The modelling framework is applied to Ghana's Bui Dam to illustrate the economy-wide impacts of a national scale energy project in a developing country. Fig. 3 shows the location of the Bui Dam and the main features of the study area. The Bui Dam is located on the Volta River in Ghana. The Volta River Basin is in West Africa and is geographically shared between six countries, with over 80% of the basin area in Burkina Faso and Ghana (Mul et al., 2015). The Volta River has three main tributaries: the Black Volta, the White Volta, and the Oti River. The construction of the Bui dam started in 2009 and hydropower generation was commissioned in 2013 with a power generation capacity of 400 MW. The total financial cost of the Bui Dam is estimated at 622 million USD and was financed through foreign loans and public spending. The Akosombo Dam, located downstream from the Bui Dam, is the largest hydropower plant in Ghana and the Volta Basin, with a power generation capacity of 1020 MW.

### 3.2 Economy-wide modelling of the Bui Dam

To analyze the economy-wide impacts of construction, financing, and operation of the Bui Dam, InvestCGE was calibrated using a disaggregated version of the 2015 Social Accounting Matrix (SAM) of Ghana (GSS, 2017). The SAM separates electricity generation into hydropower, i.e., from dams, and non-hydropower from other technologies. The costs structure used for this disaggregation is reflective of the operation costs of hydro and non-hydropower technologies. Therefore, all fuel inputs to the electricity sector were attributed to the operation of non-hydropower technologies, while the remainder of cost categories were divided proportionally between hydro and non-hydro such that the value of total inputs plus taxation across the two generation technologies is in line with the total generation data for 2015 from ECG (2017). The SAM was then translated from 2015 to 2009, the start year of the construction of the Bui Dam, by using a scaling factor of 0.528 reflecting the ratio between the GDP in the two years at constant prices. Table S1 in the supplementary material shows the input-output structure of the 2009 SAM used in model simulations.

The model was specified as dynamic-recursive running in the 2009–2067 period, with labour and capital supply being updated to follow the changes in the 18–65 age group and the investment in capital, respectively. The population changes were acquired from the Shared Socioeconomic Pathways database (O'Neill et al., 2017) and reflect the “middle of the road” SSP2 scenario. Furthermore, for agricultural land, yields are assumed to increase over time with growth rates taken from the IMPACT model (Nelson et al., 2010). The capital rents entering the hydropower cost structure were re-qualified as ‘hydropower capital rents’ and were attached to the evolution of the hydropower capital stock (dams). The hydropower capital is therefore sector-specific and cannot be re-allocated to other uses. The separation of power generation into hydro and non-hydro allows the construction of a ‘without project’ economic development baseline in which the economic system adapts to the growing demand for electricity through investment in thermal power plants. A further separation of hydropower capital assets into pre-existing assets (associated to the Akosomobo Dam) and project assets (associated to the Bui Dam) enables the identification of the government revenues from the electricity of the new dam.

Table 1 outlines the direct impacts and the main characteristics that we considered for the three project phases of the Bui Dam. The total construction cost was distributed uniformly along the construction period and was broken down as follows: 40% for construction, 23% labour, 7% services, and 30% machinery (based on a general CAPEX structure for hydropower projects from Black and Veatch, 2012). The machinery requirements, consisting mainly of turbines, were assumed to be imported while labour was considered to be locally supplied, in line with evidence from other socio-economic assessments (Kirchherr et al., 2016). The expenditure for the dam construction was done by the government and is met through the financing component of the model. As Table 1 shows, the Ghanaian government secured two foreign loans with a total of 562 million USD and provided the remaining 60 million USD from the general government budget (Dreher et al., 2017). Since the dam operates through a state-owned company, the Bui Dam capital assets were attributed to the government. Therefore, revenues from capital rents, i.e. electricity sales, were collected directly by the government and added to the general budget to cover loan repayments. For the total project-related expenditure, we assumed the government does not introduce any change to pre-existing taxation levels to raise more revenues.

**Table 1:** General illustration of the main economic characteristics of the Bui Dam.

Phase	Direct impacts
Construction	Project costs throughout the dam construction duration (4 years)
Financing	Total project finance is 622 million USD with the following breakdown: • 60 million USD from government spending • A 20-year concessional loan of 270 million USD at 2% interest • A 12-year commercial loan of 292 million USD at 1.03% over Commercial Interest Reference Rate (set at 3.5%); annual exports of 22,000 tons of cocoa at the world price over 20 years
Operation	• Reduction in electricity generation from the Akosombo Dam during initial impoundment of the Bui Dam. • Added electricity generation from the Bui Dam. • Change in electricity generation from the Akosombo Dam during the steady-state operation of the Bui Dam as a result of river flow regulation by the latter dam.

Note: GHS = Ghanaian cedi; USD = United States Dollar; 1 USD = 3.5 GHS.

Another specification that we consider in the model is the 20-year cocoa export agreement of up to 40,000 tons. Using the cocoa trade flow information for 2009–2018 from the COMTRADE database, we determined that effective trade started in 2010 with an average annual volume of 22,000 tons, or about 2.8% of cocoa production in Ghana before the Bui Dam commissioning. In this study, we thus consider this trade volume between 2010 and 2029 at an annual value of 39.6 million USD (1800 USD/ton). This agreement is implemented through an exogenous increase in government agricultural demand. The government buys the cocoa at domestic prices and exports the agreed annual volumes at the world price set in USD. The decision to follow this implementation rather than an exogenous increase in world cocoa demand passing through the Constant Elasticity of Trade (CET) exports specification of InvestCGE is also supported by the cocoa market structure in Ghana.^1^ We also assume that this additional volume of traded cocoa will not impact world prices.^2^

We do not include in this study either the economic benefits from planned irrigation water supply provided by the Bui Dam or the environmental and non-market social impacts such as population displacement. The reconciliation of non-market impacts with economy-wide modelling remains a challenge. While some attempts have been made to include environmental degradation in economy-wide modelling (e.g. Carbone and Smith, 2013), this is still at an incipient stage, notably in a developing country context. Regarding the direct benefits of electricity supply, the economic analysis in our study captures only increases in market demand and not the potential increases in social demand (obtained through higher electrification rates), nor the decrease in suppressed demand given a higher reliability of electricity supply.

### 3.3 Bio-physical system modelling

**Fig. 4:**
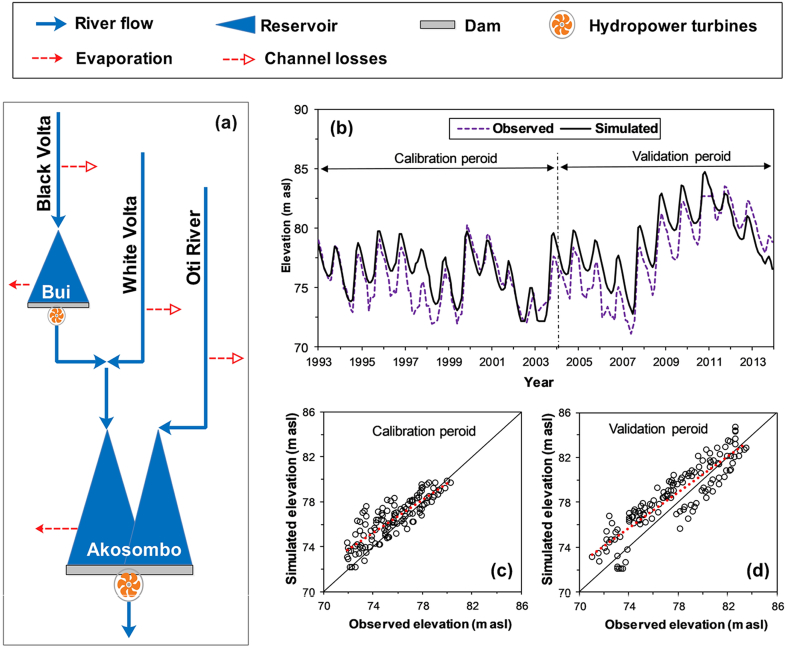
Simplified bio-physical system simulation model of the Volta River: (a) model schematic; (b) monthly time series of the Akosombo reservoir water level; (c) and (d) scatterplots of the Akosombo reservoir water level. In sub-figures c and d, the continuous and dotted lines represent the 1:1 and linear regression lines, respectively.

A simplified monthly engineered system model was developed for the Volta River to estimate electricity generation from the Bui Dam and the impact on electricity generation from the Akosombo Dam. Fig. 4a shows a schematic of the infrastructure system model. The model covers the period 1993–2013 and includes three inflow points to represent the three major tributaries of the Volta River. Monthly streamflow data for the three points were obtained from the Global Reach-Level A priori Discharge Estimates for Surface water and ocean topography (GRADES; Lin et al. (2019)). The model includes the Bui and the Akosombo dams in addition to three-channel loss points as indicated in Fig. 4a. Channel losses are accounted for at the upper parts of the Black Volta, the White Volta, and the Oti River.

The Akosombo reservoir was operated to target a constant power production while keeping the reservoir water level between the minimum operating level and the full supply level. The Bui Dam was operated similarly during the steady-state phase, i.e., after the initial impoundment. The initial filling stage of the Bui Dam was simulated based on target reservoir storage values. These target reservoir storages were derived from Landsat satellite images base on the methodology proposed by Basheer et al. (2019). This method derives a time series of reservoir surface area based on satellite imagery and transforms the areas into storages using an area-storage relationship. Fig. S1 in the supplementary material depicts the reservoir surface area of the Bui Reservoir at selected time steps.

The model was calibrated from 1993 to 2003 and was validated from 2004 to 2013. The calibration parameters included the target power of the Akosombo Dam in addition to the channel loss coefficients of the three tributaries. The performance of the model was assessed based on a quantitative comparison of the observed and simulated reservoir water levels of the Akosombo Reservoir. Water level data for the Akosombo were obtained from the Copernicus Land Monitoring Service (CGLS, 2019), which uses radar altimetry to estimate the water level of several water bodies worldwide. Three statistical performance metrics were used to assess the performance of the model. Namely, the Pearson correlation coefficient, the mean error percentage, and the Nash–Sutcliffe efficiency. Fig. 3b, c, and d depict the observed and simulated reservoir water level of the Akosombo Reservoir, and Table 2 shows the statistical performance metrics. The figures and the table reveal a good model performance. We are aware that several small-scale dams are located upstream from the spatial domain of the model. These dams were not included in the model due to the unavailability of data and assuming that they would have negligible impacts on the Akosombo Dam. This assumption is valid judging by the model performance.

**Table 2:** Model performance in the calibration and validation periods.

Performance metric	Calibration	Validation
Pearson correlation coefficient	0.84	0.9
Mean error percentage (%)	1.41	2.06
Nash–Sutcliffe efficiency	0.57	0.79

As the hydrological uncertainty was generated by sampling from historical river flow records, our approach does not consider non-stationarity in the climate system which will influence river flow in Western Africa (Roudier et al., 2014). Therefore, further work could be dedicated to understanding the operational performance of existing and planned infrastructure under different climate change scenarios.

### 3.4 Model simulations

In this study, two scenarios were considered to assess the economy-wide impacts of the Bui Dam. Both scenarios cover the 2009–2067 period, which includes 7 years of observed power generation from the Bui Dam and 50 years of water balance simulations.

The first scenario represents the baseline, in which the performance of the Ghanaian economy was evaluated assuming that the Bui Dam had not been implemented. The second scenario (‘with Bui’) includes the Bui Dam and considers the implications of construction, financing, and operation of the dam. In the ‘with Bui’ scenario, actual electricity generation data for Bui Dam were used for the period 2009 to 2016. For the same period, the impact of the Bui Dam on the flow of the Black Volta was removed using the bio-physical model. Then electricity generation from the Akosombo Dam was simulated based on the unaltered flow of the Black Volta to represent energy generation from the Akosombo Dam in the baseline scenario. For the 2017–2067 period, the calibrated bio-physical model was used to estimate electricity generation in the two scenarios under 50 plausible sets of streamflow conditions (i.e., we consider 50 possible hydrological futures assuming the variability observed historically will continue – no climate change signal). The plausible future (‘stochastic’) hydrological conditions were generated by ‘bootstrapping’ from the historic flow record (Efron, 1992), i.e., sampling from the historical hydrological years and recombining them into possible sequences of future flows.

To evaluate the significance of the economy-wide impacts of the dam construction, financing and operation, we employed three Economic Impact Variants (EIVs) for the ‘with Bui’ scenario. These EIVs are:

• ‘electricity-only’ - this case determines the economy-wide impacts of an increase in hydropower production without considering the investment and finance needs. This serves as a basic impact variant and has been the approach taken in previous impact assessments of hydropower dams, which consider only the operational stage.
• ‘loans’ - a case where, in addition to higher hydropower production, the project costs are considered during the construction phase of the dam and are covered using the two government-backed loans (12-years and 20-years) as described above. This EIV does not include the cocoa export agreement.
• ‘loans+exports’ – a similar case as ‘loans’ but includes the cocoa exports over the 2010–2028 period. The difference between the ‘loans’ and the ‘loans+exports’ EIVs provide insights into the economy-wide impacts of negotiated conditions for securing loans at preferential interest rates.

**Fig. 5:**
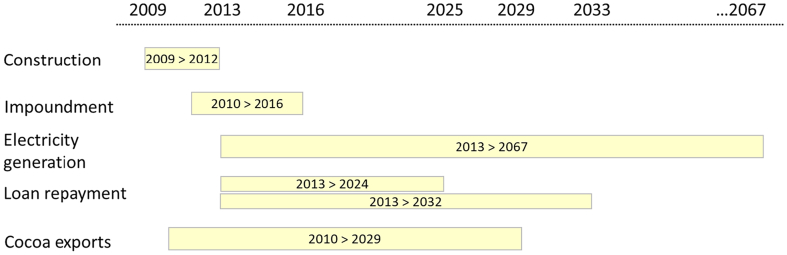
Bui Dam project timeline.

An outline of the scenarios and EIVs used in this analysis is included in Table S2 of the supplementary material while the timeline on the different project components are presented in Fig. 5. The modelling results are reported in the next section as deviations of macro-economic, sector- and household-level metrics of the three EIVs relative to those obtained in the baseline for the 2009–2067 timeframe.

## 4 Results

### 4.1 Macro-economic impacts

**Fig. 6:**
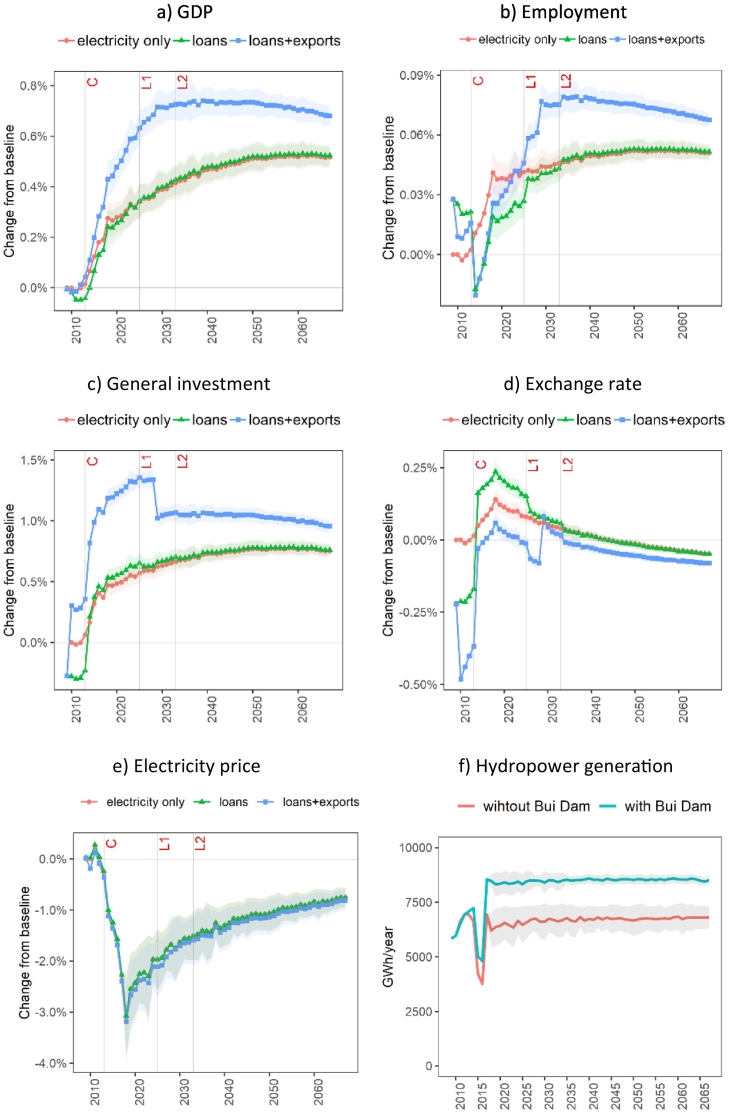
Impacts of construction, financing, and operation of the Bui Dam on the Ghanaian macroeconomic variables (a) real GDP, (b) employment, (c) general investment, (d) exchange rate, and on the electricity market through changes in (e) electricity prices, and (f) hydropower generation. Points represent the mean and ribbons indicate the standard deviation of the results due to hydrological uncertainty. Note: C = construction completion; L1 = maturity of first loan; L2 = maturity of second loan.

Fig. 6a-d depict the modelled macro-economic impacts of the Bui Dam. Looking at the ‘electricity only’ EIV in Fig. 6a, the results show that excluding project requirements for construction and financing, the Bui Dam, once in operation, would on average induce an increase of 0.18% in GDP during the initial impoundment period (by 2016), and 0.55% in the long-term. The increase in hydropower generation (Fig. 6f) would lead to positive effects throughout the economy by increasing the output of non-energy sectors, exports of commodities, employment^3^ (Fig. 6b), and household income and expenditure. The increase in electricity supply reduces the electricity generation cost, which translates into a reduction of electricity price (Fig. 6e), and accelerates the Ghanaian GDP growth relative to the baseline with higher levels of private savings and investment (Fig. 6c) facilitating this economic expansion.

Fig. 6a also reveals that the decisions around the construction and financing of the Bui Dam would affect the Ghanaian GDP throughout the lifetime of the dam, i.e., during construction (2009–2012), loan repayment (2013−2032), and beyond. The ‘loans’ EIV shows a decline in GDP for the construction period driven by two factors: (1) a decrease in non-project economic activities due to a crowding-out effect in the labour and internal capital market and (2) an increase in investment costs outside project construction. The impacts on GDP during the construction phase drop to −0.05% in 2013, relative to the baseline. Nevertheless, these impacts are considerably smaller than the annual project costs.

After the operation start of the Bui Dam in 2013, GDP shows a slightly higher rate of increase in the ‘loans’ EIV than in the ‘electricity only’. Though starting from a lower GDP point in 2013, ‘loans’ outperforms ‘electricity only’ in GDP growth during the repayments of the first loan (between 2013 and 2024) as economic activity is stimulated through higher exports enabled by an increase in the exchange rate. In fact, the loan repayments in foreign currency lead to a depreciation of the domestic currency (Fig. 6d) making domestic commodities less expensive for export markets.

The addition of the cocoa exports (‘loans+exports’ EIV) increases GDP from 2010 when the export agreement becomes operational. Most of this economic growth is driven by an increase in agricultural output (as cocoa production is part of this sector), which leads to a positive effect on investment (Fig. 6c). However, the increase in cocoa exports leads to an appreciation of the local currency and therefore it generates a negative impact on other exports. Nevertheless, the net effect on output for most economic sectors are positive in the long run (see Section 4.2). After the cocoa export agreement ends in 2028, the GDP gains decline as the economy re-adjusts to the lower levels of agricultural demand. In the same year, there is also an immediate impact on employment (Fig. 6b) driven by higher public expenditure as the government's export-related cocoa procurement expenditure ends (see government account impacts in Section 4.3.3). Simultaneously, the depreciation of the local currency after 2028 would enable higher levels of exports, which stimulate economic activity outside agriculture.

During the construction phase (2009–2012), total employment shows a slight increase of around 0.03% in the ‘loans’ EIV (Fig. 6b) with most of the project direct and indirect labour requirements sourced from the other sectors (see Section 4.3). In contrast, in ‘loans+exports’ total employment is reduced as the additional agricultural demand for exports increases the general cost of labour and thus lowers labour demand from the other economic activities.

The project demand for construction services in the ‘loans’ EIV increases the cost of investment in other sectors of the economy and reduces general (non-project) investment (Fig. 6c). The cocoa exports in ‘loans+exports’ offset this effect through an increase in income for rural households and a consequent increase in investment in agricultural activities.

Due to foreign loan repayments, the local currency in the ‘loans’ EIV would depreciate across the 12-year and 20-year periods over which the two loans are repaid (Fig. 6d). In this timeframe, two marked changes in the exchange rate occur, in 2025 and 2033 when the first and second loans respectively reach their maturity.

The introduction of variable hydropower generation slightly changes the time profile of these macro-economic impacts (see Fig. S2 in Supplementary Material for macro-economic impacts using deterministic hydropower generation). The standard deviation of GDP and employment values across the 50 streamflows is higher than the differences between the mean values in the ‘electricity-only’ and ‘loans’ EIVs, but much smaller than the mean differences between these two EIVs and ‘loans+exports’. For GDP, the standard deviation is 0.07% across the three EIVs in 2018 and declines to 0.04% towards the end of the simulation period.

### 4.2 Sectoral production

**Fig. 7:**
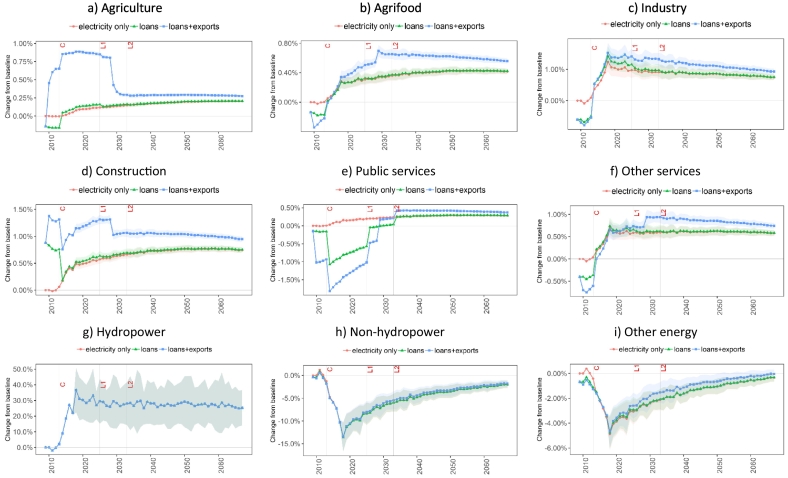
Impacts of construction, financing, and operation of the Bui Dam on the Ghanaian sectoral production. The points represent the mean and the ribbons indicate the standard deviation of the results due to hydrological uncertainty. Note: C = construction completion; L1 = maturity of first loan; L2 = maturity of second loan.

Fig. 7 shows the impact of the Bui Dam on sectoral output across thre three EIVs considered. During the project construction phase, most economic activities face a slight reduction in output (‘loans’ EIV) due to the crowding-out of capital and labour to meet the project financing and workforce requirements. The only exception is the construction sector which is boosted by 0.9% during the construction period (2009–2012) given its direct inputs in this phase of the project (Fig. 7d). At the same time, the activity levels in services and industry show a decrease of 0.4% and 0.6%, respectively, due to the economy-wide increase in wage levels induced by a higher labour demand in construction.

The increase in the availability and lower price of electricity in the ‘electricity only’ EIV has a positive impact on output across all economic activities. Industry and services have the highest increase in activity levels (1.1% and 0.6%, respectively) after the end of the dam impoundment in 2016. The difference in the increase in activity levels across sectors is determined by the energy intensity of each sector and the ability to substitute other energy inputs with electricity. However, as expected, sectors with higher electricity dependency are more vulnerable to hydrological uncertainty. This can be observed in the standard deviation of output in services and industry (Fig. 7f and c, respectively), which is higher than in other sectors. Hydrological uncertainty could therefore increase or decrease production by 0.1–0.3% and 0.1–0.2% in industry and services, respectively, relative to the baseline.

The inclusion of loan repayments (‘loans’ EIV) leads to a contrast between the impacts on the private and public sectors. Public services (health, education and public administration) (Fig. 7e), being funded by the government, face a decrease of 1.5% in output in the first year after dam operation given that the government pays for the difference between the sales of generated project electricity and loan repayments (see Section 4.3.3). Loan repayment in foreign currency results in an increase in the exchange rate. Therefore, outside the public sector, during the 2013–2032 period, all the other activities show an increase in output compared to the ‘electricity only’ EIV due to an increase in export volumes enabled by a domestic currency depreciation (see commodity export changes in Fig. S3 in Supplementary material).

The ‘loans+exports’ EIV further influences the distribution of impacts across sectors. Given that cocoa production is included in the agriculture sector (Fig. 7a), the demand for cocoa exports leads to a further increase in agricultural production but also to higher prices of agricultural commodities as agricultural output is constrained by land availability. In fact, the increase in demand for cocoa rises land rents and increases the costs and market prices of agricultural commodities. This price increase affects the agrifood industry negatively (Fig. 7b). The output levels of industry (until 2018) (Fig. 7c) and other services (until 2022) (Fig. 7f) are also lower compared to the ‘loans’ EIV, due to the re-allocation of capital and labour towards agriculture. The export agreement also affects public spending, as the government compensates cocoa producers for the difference between the world market price and the domestic market price. Therefore, public services see a decline in activity output with a cutback of 1% at the start of the export agreement in 2010 (Fig. 7e).

Across the three EIVs, despite an initial average increase of 26.3% in hydropower output (representing 13.4% of total pre-Bui total electricity generation), only 3.2–3.6% additional electricity is absorbed domestically by 2016. This is due to hydropower production displacing some of the non-hydro electricity generation technologies in the first years of dam operation. In fact, the additional production capacity lowers electricity prices and pushes part of the non-hydropower generation out of the market by 2018 (Fig. 7h). A similar yet smaller effect takes place between electricity and other energy commodities (Fig. 7i) as electricity is preferred over other energy forms given its lower consumer price. In the long-term, the use of non-hydropower and that of other energy commodities gradually recovers to the baseline values as the economy expands and requires more energy to support the production and consumption of goods and services.

### 4.3 Social impacts

#### 4.3.1 Sectoral employment

**Fig. 8:**
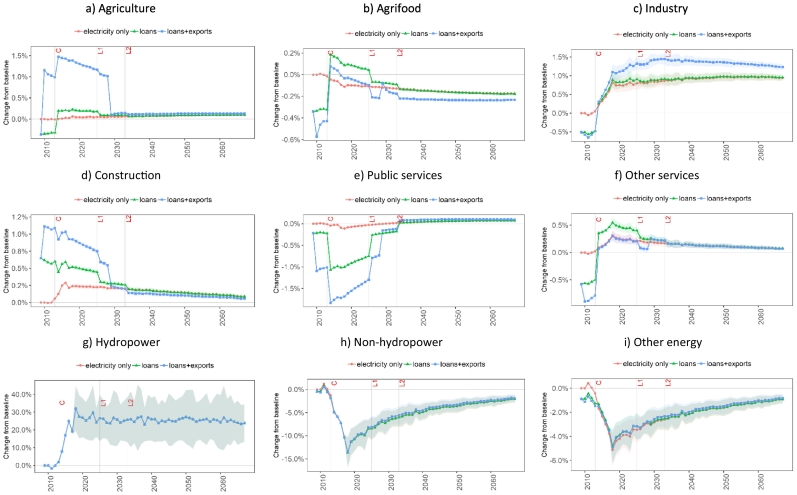
Impacts of construction, financing, and operation of the Bui Dam on the Ghanaian sectoral employment. The points represent the mean and the ribbons indicate the standard deviation of the results due to hydrological uncertainty. Note: C = construction completion; L1 = maturity of first loan; L2 = maturity of second loan.

Fig. 8 shows the impacts of the Bui Dam on sectoral employment across the three EIVs. During the construction phase, construction is the only sector with an increase in employment. Other sectors suffer a crowding-out effect leading to a decrease in employment in the range of 0.3–0.8%. After the initial filling of the dam, the ‘electricity-only’ EIV shows an increase in employment in the industry, services, and construction sectors, and a decrease in employment in the agrifood sector. The impact on employment in the different sectors depends on their labour intensity (units of labour used per unit of output) and the ability of electricity to increase labour productivity.

During the operation phase, loan repayments (‘loans’ EIV) negatively affect total employment despite an overall increase in economic activity reflected by higher GDP levels. As shown in Fig. 8, employment levels increase in many non-public sectors (agriculture, industry, construction and other services) driven by a higher output; however, the reduction in activity in public services results in a net decrease in total employment on the short term.

By adding the cocoa export agreement (‘loans+exports’ EIV), total employment decreases more compared to the ‘loans’ EIV, but it recovers after 2020. This overall reduction is due to a further decrease in labour demand in public services, agrifood and other services. Higher cocoa exports have a positive impact on employment only in agriculture (due to an increase in demand for cocoa), construction (due to higher investment in agricultural capital) and industry (due to a higher demand of intermediate inputs in the agricultural sector). The decrease in employment in agrifood in conjunction with an increase in output in this sector (Fig. 7b) highlights a substitution of labour with capital. Due to the cumulated investment in the sector, this substitution has a long-term effect as the labour requirements in 2030 and after, when the cocoa export agreement expires, are below the baseline levels while output levels are above.

In line with the sectoral output results from above, hydrological variability mostly impacts employment related to energy production (Fig. 8g-i) – standard deviations of 8.1–20.8%, 0.8–3.3% and 0.3–1.5% are observed in hydropower, non-hydropower and other energy, respectively. Hydropower generation uncertainty only has small effects on labour demand in industry and services with standard deviations of 0.05–0.1%.

#### 4.3.2 Household income and consumption

**Fig. 9:**
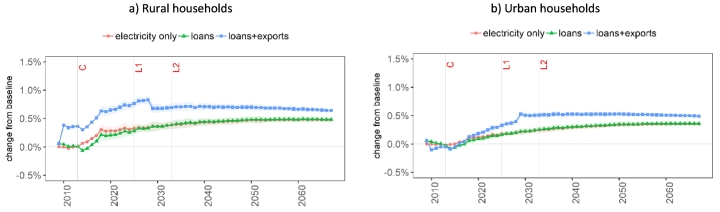
Impacts of construction, financing, and operation of the Bui Dam on consumption expenditure of Ghanaian households. The points represent the mean and the ribbons indicate the standard deviation of the results due to hydrological uncertainty. Note: C = construction completion; L1 = maturity of first loan; L2 = maturity of second loan.

Fig. 9 shows the impact of the Bui Dam on the Ghanaian household welfare measured as a change in household consumption expenditure for rural and urban households. The project construction phase in the ‘loans’ EIV shows a slightly positive impact on the two household groups in the EIVs that include the project construction requirements. The increase in hydropower generation alone (‘electricity only’) resulting in higher employment in labour-intensive sectors (agriculture, industry and construction) has a slightly higher impact on the income and expenditure of the rural households compared to that of urban households. Compared to ‘electricity only’, the ‘loans' EIV shows a reduction in the income and expenditure of all households during the dam impoundment phase and only a negligible effect in the long run. Both ‘electricity only’ and ‘loans' EIVs show an increase in income over time.

The cocoa export agreement (‘loans+exports’) affects both household groups in a more significant manner. The urban households see a reduction in consumption expenditure up to 2018 given lower revenues from employment and non-agricultural capital. At the same time, rural households increase their revenues due to higher agricultural production and higher land rents. In 2029, at the end of the export agreement, the income and expenditure of rural households decrease contrarily to urban households. This contrast is explained by the re-orientation of government expenditure towards public services.

Fig. 9 shows only a slight impact of hydrologic uncertainty on household income and expenditure. The standard deviation of income due to uncertain hydropower generation is small for both household groups across all three EIVs, with values in the range of 0.13–0.22 USD/capita/year.

#### 4.3.3 Government income and expenditure

**Fig. 10:**
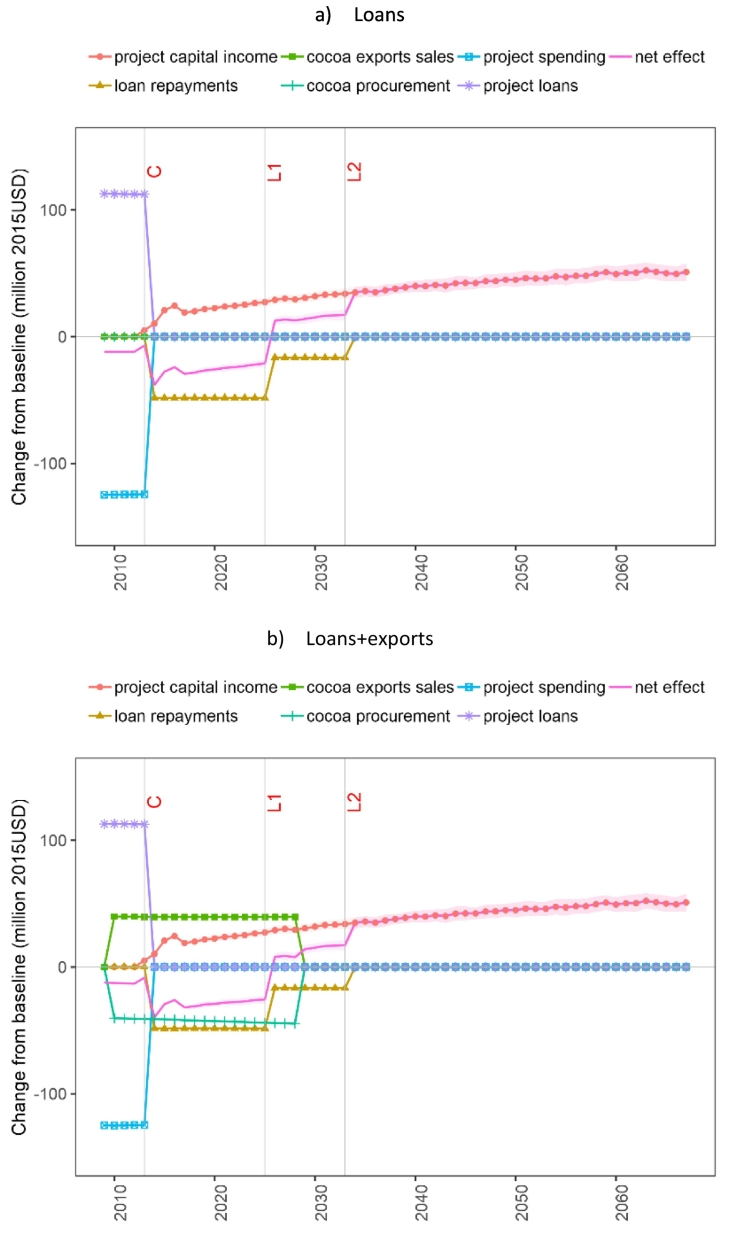
Changes in government income and expenditure related to project financing and operation. Points represent the mean values and the ribbons indicate the standard deviation of the results due to hydrological uncertainty. Note: C = construction completion; L1 = maturity of first loan; L2 = maturity of second loan.

Fig. 10a-b illustrate the income and expenditure implications for the government in the ‘loans’ and ‘loans+exports’ EIVs. The government's accounts are identically impacted during the construction phase as the project loans and project spending are the same. Also, during the operation phase, the project-related income from loans, capital income from Bui electricity sales, and the loan repayments are similar across the two EIVs.

Since sales of electricity generated through the project are initially zero and gradually increase over time, the government faces higher loan repayments than returns from electricity sales during the first 16 years of the dam operation. Therefore, the net effect over the government accounts is negative (with a net increase in expenditure of 21–38 million USD/year) until the end of repayments of the first loan (Fig. 9a), and then becomes positive due to both higher electricity revenues and lower repayment levels (with a net increase in annual revenues of 12.5 million USD by 2025).

The export agreement (‘loans+exports’) increases the government's annual expenditure by between 0.5 million USD (in 2010) and 5 million USD (in 2028). This increase occurs because the cost of cocoa procurement is higher than the income from cocoa exports which is set at a fixed world price (Fig. 9b). This procurement expenditure results in a reduction in spending on public services (Section 4.2).

Hydrological uncertainty plays a minor role in the net effect on government accounts. Only capital income from Bui electricity sales has a noticeable standard deviation of 2–6 million USD/year.

### 4.4 Cumulated economic impacts

**Fig. 11:**
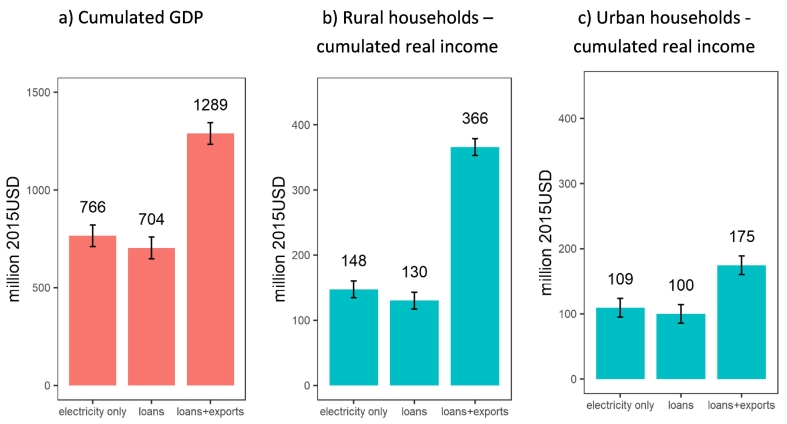
Cumulative impacts during the 2009–2067 period: (a) GDP gains (b) real income of rural households and (c) real income of urban households. Annual values are cumulated using a 10% discount rate. The columns and the numbers above the columns indicate the mean. Whiskers represent the standard deviation of cumulative impacts across the 50 simulated river flow sequences.

Under hydrological variability, the total mean cumulative GDP impacts in the 2009–2067 period calculated at a 10% discount rate are 766 million USD, 704 million USD and 1289 million USD for the ‘electricity-only’, ‘loans’ and ‘loans+exports’ EIV, respectively (Fig. 11a). To put these values in perspective, they are comparable or considerably higher than the initial value of the project (622 million USD). The standard deviation is similar across the three EIVs at 55 million USD, and a coefficient of variation of 7.2%, 7.8% and 4.3% of the mean values for the ‘electricity-only’, ‘loans’ and ‘loans+exports’ EIV, respectively.

At the household level, the cumulative real income^4^ effect is higher for rural households than for urban households (Fig. 11b-c). The largest differences between the two groups are obtained in the ‘loans+exports’ EIV since the increase in agricultural output has a strong effect on the income of rural households – 366 million USD median for rural households and 175 million USD median for urban households. The standard deviation due to hydrological variability is about 14.3 million USD and 12.8 million USD for rural and urban households, respectively.

The levels of cumulative effects depend on the choice of discount rate. Table 3 includes the GDP results of a sensitivity analysis using alternative discount rates, including cumulative values without any discounting. As expected the sensitivity analysis shows that mean values expand with lower discount rates. Nevertheless, even with a zero discount rate, the ‘loans+exports’ EIV leads to a 47.7% higher mean GDP value than ‘electricity only’ and to a coefficient of variation due hydrological variability of 4.1% (against 6.1% in ‘electricity only’). For the ‘loans’ EIV, a lower discount rate increases the value of the project in GDP terms relative to the one for ‘electricity only’.

**Table 3:** Cumulated GDP sensitivity analysis on discount rate choice.

EIV	Discount rate	Mean *USD2015 million*	Mean relative to ‘electricity only’ mean	SD *USD2015 million*	Coefficient of variation
Electricity only	0%	14,671.62	100.0%	892.22	6.08%
	3%	4853.83	100.0%	306.72	6.32%
	5%	2592.95	100.0%	170.47	6.57%
	10%	765.15	100.0%	55.84	7.30%
Loans	0%	14,785.06	100.8%	892.39	6.04%
	3%	4827.63	99.5%	306.78	6.35%
	5%	2539.98	98.0%	170.50	6.71%
	10%	703.21	91.9%	55.84	7.94%
Loans + exports	0%	21,840.01	148.9%	892.94	4.09%
	3%	7545.67	155.5%	307.01	4.07%
	5%	4146.63	159.9%	170.65	4.12%
	10%	1288.52	168.4%	55.92	4.34%

## 5 Conclusions and policy implications

This study introduced a coupled CGE and a water system modelling framework to quantify the economy-wide impacts of hydropower development across project construction, financing, and operation. The framework was applied to the 400 MW Bui Dam in Ghana requiring an investment of 622 million USD. The project was financed through concessional loans enabled by a cocoa export agreement with China. Results demonstrate that the inclusion of the construction and financing phases, as well as the consideration of other macro-economic drivers (factor supply, exchange rates and public spending) through an economy-wide assessment, changes both the cumulative value and the temporal evolution of the economic gains and losses of large infrastructure projects.

The inclusion of the financing conditions for the Bui Dam leads to a 68% increase in mean GDP gains at a discount rate of 10% (from 766 million USD in the ‘electricity only’ variant to 1289 million USD in ‘loans+exports’), driven by the additional economy-wide benefits of higher cocoa production. Hydrological variability impacting electricity generation introduces a standard deviation of the discounted GDP gains of only 7.3% (‘electricity only’), 7.9% (‘loans’) and 3) and 4.3% (‘loans+exports’). These results, therefore, support the literature claiming that an economy-wide approach that goes beyond the quantification of the direct impacts of projects is appropriate for ex-ante economic assessments of infrastructure investments.

Despite the reliance of the Ghanaian economy on cocoa exports, the export agreement in combination with the dam commissioning does not yield Dutch disease effects.^5^ Indeed, the higher exports determine a re-allocation of labour towards agriculture, however, the effect is partial since only agrifood and public services employment decrease while output in agrifood exceeds baseline levels. Employment in industry and construction increases given higher investment and income levels. At the same time economic activity across all sectors, with the exception of public services, is positively impacted, with the higher electricity supply being an important driver.

The results of the EIVs considering the construction and financing project components also capture crowding out effects related to government resources. One relevant case is that of public services for which the output is reduced by 1–1.7% during the loan repayment period. This lower government spending could have further impacts on education and healthcare provision with longer term effects on labour productivity. Although a direct relationship between public spending and productivity is difficult to endogenise in the absence of empirical evidence, this effect could reduce the benefits on output, GDP and welfare obtained in this study.

The assessment of the economic impacts of the Bui Dam used actual project data for 2009–2016, thus eliminates uncertainties related to construction cost, financing, and hydrological flows during this period. In more conventional cases, the framework can be used to inform policymakers in ex-ante analyses to study the potential economic impacts of financial options, loan conditions and electricity production vulnerabilities. The framework could be employed to explore the viability of alternative financing sources with different interest rates and with alternative mechanisms for budgetary compensation of loan repayments. In our assessment, it was assumed that loan repayments and the cocoa collateral price compensation by the government are made through deductions from the government's general budget without any changes to the pre-existing tax regime. Ex-ante analyses could also consider the impact of potential changes to fiscal policies to cover project-related costs.

The CGE model used in the present study includes an advanced specification of government income and expenditure accounts but does not consider a behaviour where investment agents would seek to maximize investment yields as specified in financial CGE models (see Robinson (1991) for a discussion on including financial assets in a CGE framework). Nevertheless, the assumption of yield maximization for foreign lenders in developing countries should be brought under scrutiny considering that interest rates of foreign loans may be negotiated based on objectives other than direct financial returns. The Bui Dam financing is an example of preferential rates obtained in exchange for cocoa exports, which may address China's concerns related to the resource's security of supply.

In spite of the commissioning of the Bui Dam, the Ghanaian electricity sector continues to be affected by power outages which have a negative economic impact of businesses and households. While peak load capacity increased, the domestic electricity demand continued to outstrip supply. At the same time, on the supply side, the low flows in the Volta basin in the 2014–2016 period, embedded in the simulations of this study, reduced hydropower output. Furthermore, the electricity system in Ghana is impacted by other issues such as natural gas supply unreliability affecting combustion power plants, significant losses in the electricity distribution networks and difficult access to finance for additional capacity (Kumi, 2017). Consumers cope with the intermittencies resulting from the supply-demand imbalances by investing in back-up generation and moving economic activity to areas or times of day with more reliable supply (Owusu et al., 2022). These coping mechanisms induce an economic cost which was partially considered in this study. By introducing alternative power technologies our modelling framework, the use of back-up technologies was implicitly considered in both the baseline and the scenarios with the Bui Dam commissioned. Furthermore, through the addition of the Bui Dam, the model simulations captured a reduction in generation costs and market prices of electricity. This price effect depicts a lower reliance on higher-cost back-up generation technologies compared to the baseline. Nevertheless, other negative effects of intermittency such as impacts on production quality highlighted in Owusu et al. (2022) were not included due to lack of quantifiable information. Furthermore, while there have been efforts by others to include intermittent power supply in a top-down modelling frameworks such as CGE which runs in annual time steps (Tapia-Ahumada et al., 2015), an integrated approach adopting energy models with a high spatial and temporal resolution may be more suitable for such exercises. We leave this integration for future work while acknowledging that the benefits of lower intermittency measured through specific system reliability metrics due to the Bui Dam were not fully considered in this study.

The cocoa export volumes in this study were considered to be constant at 22,000 tons and 1800 USD/ton. An inspection of cocoa trade flows for 2010–2018 using the COMTRADE database reveals that both the annually exported volumes and the trade prices between Ghana and China have fluctuated significantly. At this point, we can speculate that these variations could be attributed to changes in export demand from China and domestic supply levels in Ghana. Therefore, the inclusion of trade variability could add another level of complexity to the present analysis.

Given that this study covers both past and future periods, it could be relevant to compare the results obtained here with actual macro-economic and sectoral indicators from 2009 onwards. We hold back from carrying out such comparison as other larger economic shocks have taken place in the Ghanaian economy during the past decade, notably in relation to the oil and gas industry (Gyimah-Boadi and Kwasi Prempeh, 2012; Ayelazuno, 2014), which means it would be difficult to identify and isolate the specific economic signals of the Bui dam in historical economic indicators. Going forward, the analysis baseline and simulations do not include specific structural changes which could occur in the economy over the long time horizon considered. The InvestCGE framework allows for the inclusion of these changes through model parameter recalibration between simulation time steps. To illustrate this model feature the results of an alternative specification allowing for the demand system dynamic recalibration is displayed in Fig. S4 in the Supplementary Material.

While climate change effects on hydrological uncertainties have not been included in the analysis, we acknowledge their importance in project evaluation. This is applicable notably for the accounting of economic benefits of hydropower generation expecting mean annual electricity production to change and variability to increase in the coming decades. Nevertheless, the sensitivity analysis on discount rates shows that even at a 0% rate which increases the weight of long-term effects in the project present value calculation, the project financing conditions included in the ‘loans+exports’ EIV still have an important impact on cumulated GDP effects.

Our analysis highlights that loan conditions with collateral are as important as the economy-wide net benefits of increased energy infrastructure service levels. Non-traditional lenders such as the Chinese Government have a growing stake in providing project finance in developing countries (Foster and Briceño-Garmendia, 2009; Horn et al., 2019), with objectives that often diverge from investment yield maximization. In this changing landscape of infrastructure financing, our results endorse the argument that a comprehensive consideration of investment conditions in the evaluation of the benefits of infrastructure investment is relevant for economy-wide development analyses.

## CRediT authorship contribution statement

**Victor Nechifor:** Conceptualization, Data curation, Formal analysis, Investigation, Methodology, Validation, Visualization, Writing – original draft, Writing – review & editing. **Mohammed Basheer:** Conceptualization, Data curation, Formal analysis, Investigation, Methodology, Validation, Visualization, Writing – original draft, Writing – review & editing. **Alvaro Calzadilla:** Conceptualization, Methodology, Validation, Writing – review & editing, Funding acquisition. **Emmanuel Obuobie:** Data curation, Writing – review & editing. **Julien J. Harou:** Conceptualization, Validation, Writing – review & editing, Funding acquisition.

## Declaration of Competing Interest

None.

## Appendix A Supplementary data

Supplementary material Image 1

InvestCGE GAMS model Image 2

## Footnotes

1 Cocoa exports are a government monopoly through the Ghana Cocoa Board, the COCOBOD. Exports are thus not liberalized as COCOBOD buys from the farmers prices established prior to the harvesting season and then sells on the world market (Kolavalli and Vigneri, 2018). The assumption in the paper is that COCOBOD will buy the additional export requirements and will bear the costs of differences between market prices and changes to farmgate prices due to the new demand. According to Boysen et al. (2021), COCOBOD imposes a ‘policy wedge’ whereby farmgate prices are about 30% lower than the buyer prices, leaving enough flexibility for government to absorb the difference between buying and selling prices.

2 Ghana produces about a fifth of world cocoa production, but other producing countries e.g. Côte d'Ivoire (representing two fifths of world cocoa production) could displace Ghana's production, now at a higher marginal cost. Furthermore, the analysis in Boysen et al., 2021 shows that even when the two top producing countries (Côte d'Ivoire and Ghana) aim to increase world cocoa prices through e.g. export levies, this is partially offset by production diversion to other countries. Therefore, we assume that through this trade arrangement (representing 0.5% of world cocoa production) the export price of Ghanaian cocoa does not diverge from the world price to not affect the overall traded volume by the country.

3 Given the endogenous labour supply in the InvestCGE specification, the increase in employment involves an increase in aggregate labour supply with full employment.

4 The real income is measured through the equivalent variation, which is a standard measure used in welfare economics to determine the net income effects for households when considering changes in consumer price.

5 The Dutch disease refers to the negative general equilibrium effects of the expansion in exports of a group of commodities. The effect is largely attributed to an appreciation of the real exchange rate making other sectors less competitive in the domestic and international markets.
