# Supplementary material for: Financing national scale energy projects in developing countries – An economy-wide evaluation of Ghana's Bui Dam
Source: Energy Econ. 2022 Jul;111:None. doi: 10.1016/j.eneco.2022.106065 (PMC9240999; doi:10.1016/j.eneco.2022.106065)
Supplement: Supplementary file 1 — Supplementary material [file mmc1.docx]

Supplementary material

Table S1 Input-output structure of the Ghana SAM for 2009.

|  | **Agriculture** | **Industry** | **Agrifood** | **Construction** | **Transport** | **Non-hydro electricity** | **Hydro electricity** | **Other energy** | **Services** | **Public services** | **Rural households** | **Urban households** | **Government** | **Investment** | **Stocks** | **Transport margins** | **Exports** | **Total** |
| --- | --- | --- | --- | --- | --- | --- | --- | --- | --- | --- | --- | --- | --- | --- | --- | --- | --- | --- |
| **Agriculture** | 329.4 | 87.0 | 3360.1 | 0.0 | 0.0 | 0.0 | 0.0 | 0.0 | 695.1 | 0.0 | 4141.3 | 4605.6 | 0.0 | 0.0 | 222.8 | 0.0 | 2918.2 | 16359.5 |
| **Industry** | 758.0 | 10890.3 | 887.2 | 4031.7 | 3173.5 | 385.8 | 1325.1 | 1187.9 | 3189.0 | 2296.5 | 1860.1 | 5159.0 | 0.0 | 5049.1 | 0.0 | 7885.2 | 13175.2 | 61253.6 |
| **Agrifood** | 488.9 | 1530.2 | 626.1 | 0.0 | 41.0 | 9.5 | 22.0 | 8.3 | 1636.7 | 351.6 | 4540.1 | 7928.1 | 0.0 | 0.0 | 470.6 | 0.0 | 995.8 | 18649.0 |
| **Construction** | 62.3 | 52.5 | 7.7 | 18.4 | 0.0 | 0.0 | 0.0 | 0.0 | 365.7 | 371.8 | 61.0 | 143.1 | 0.0 | 13896.1 | 0.0 | 0.0 | 0.0 | 14978.4 |
| **Transport** | 52.2 | 5171.4 | 268.5 | 17.1 | 327.0 | 45.5 | 147.7 | 36.1 | 313.6 | 396.6 | 476.1 | 1496.3 | 0.0 | 0.0 | 0.0 | 3968.2 | 957.7 | 13673.9 |
| **Electricity** | 50.1 | 1268.4 | 152.8 | 0.0 | 27.0 | 37.3 | 119.0 | 116.0 | 608.4 | 353.7 | 286.6 | 1529.4 | 0.0 | 0.0 | 0.0 | 0.0 | 4.4 | 4553.1 |
| **Other energy** | 0.0 | 2375.1 | 0.0 | 0.0 | 0.0 | 1549.8 | 0.0 | 0.0 | 0.0 | 0.0 | 0.0 | 0.0 | 0.0 | 0.0 | 657.9 | 0.0 | 1973.2 | 6556.0 |
| **Services** | 102.2 | 3731.4 | 107.4 | 0.0 | 342.9 | 108.4 | 370.1 | 587.5 | 6287.9 | 2729.9 | 3167.4 | 9033.8 | 0.0 | 0.0 | 0.0 | 0.0 | 8942.2 | 35511.1 |
| **Public services** | 262.4 | 0.0 | 4.7 | 0.0 | 218.5 | 0.0 | 0.0 | 0.0 | 18.3 | 985.3 | 476.6 | 1891.7 | 11788.7 | 0.0 | 0.0 | 0.0 | 258.3 | 15904.6 |
| **Taxes and tariffs** | 2775.9 | 9434.3 | 6403.5 | 0.0 | 6.9 | 2.2 | 2.2 | 678.2 | 509.0 | 12.2 |  |  |  |  |  |  |  |  |
| **Labor** | 4208.2 | 3134.5 | 731.2 | 1367.9 | 2580.8 | 14.0 | 37.5 | 747.1 | 4511.5 | 5459.8 |  |  |  |  |  |  |  |  |
| **Capital - agriculture** | 2207.9 | 0.0 | 0.0 | 0.0 | 0.0 | 0.0 | 0.0 | 0.0 | 0.0 | 0.0 |  |  |  |  |  |  |  |  |
| **Capital - other** | 0.0 | 4567.8 | 2441.8 | 9543.4 | 4983.5 | 84.8 | 0.0 | 2605.4 | 8769.1 | 1556.6 |  |  |  |  |  |  |  |  |
| **Capital - hydropower** | 0.0 | 0.0 | 0.0 | 0.0 | 0.0 | 0.0 | 287.7 | 0.0 | 0.0 | 0.0 |  |  |  |  |  |  |  |  |
| **Land** | 4424.7 | 0.0 | 0.0 | 0.0 | 0.0 | 0.0 | 0.0 | 0.0 | 0.0 | 0.0 |  |  |  |  |  |  |  |  |
| **Imports** | 637.3 | 19010.7 | 3657.9 | 0.0 | 1972.8 | 2.2 | 2.3 | 589.4 | 8606.8 | 1390.8 |  |  |  |  |  |  |  |  |
| **Total** | 16359.5 | 61253.6 | 18649.0 | 14978.4 | 13673.9 | 2239.5 | 2313.5 | 6556.0 | 35511.1 | 15904.6 |  |  |  |  |  |  |  |  |

*Note: All the values are in millions of 2015 Ghanaian Cedi (GHS)*

Table S2 Scenario and EIV outline

| **Scenario** | **Socio-economic development** | **EIV** | **Hydropower generation dynamics** |
| --- | --- | --- | --- |
| Baseline | Population and labour supply changes based on SSP2  Endogenous capital accumulation | n/a | Hydropower generation without the Bui Dam from calibrated water balance model:  Historical hydrological conditions for 2009-2016  Stochastic hydrological conditions for 2017-2067 |
| With Bui |  | Electricity-only | Hydropower generation with the Bui Dam from calibrated water balance model:  Historical hydrological conditions for 2009-2016  Stochastic hydrological conditions for 2017-2067  + Deterministic hydropower generation equal to the annual mean generation of the 50 stochastic streamflow conditions for 2017-2067 |
|  |  | Loans |  |
|  |  | Loans+exports |  |


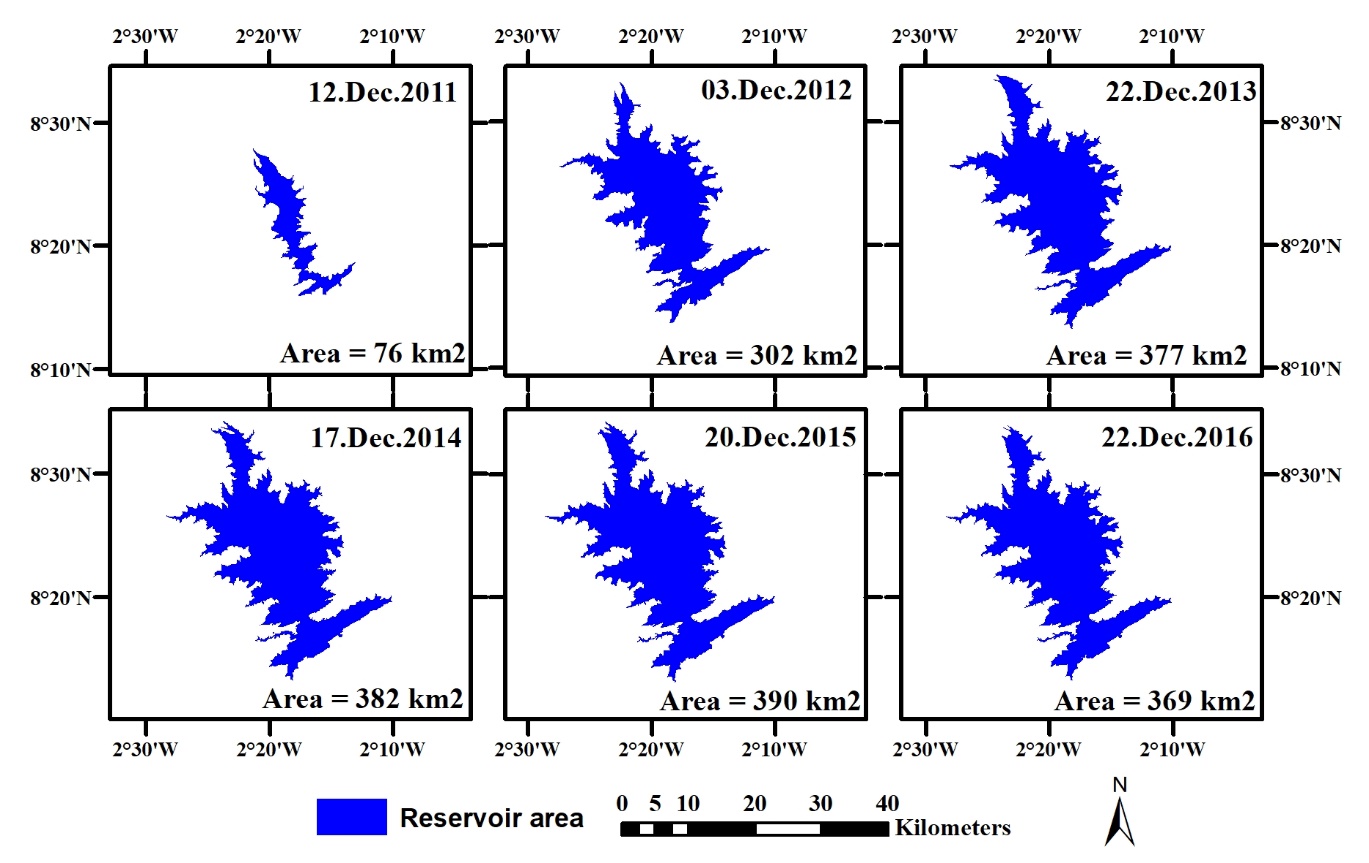


Fig. S1 Surface area of the Bui Reservoir at selected time steps. The displayed data are based on Landsat satellite images.

| a) GDP | b) Employment |
| --- | --- |
| 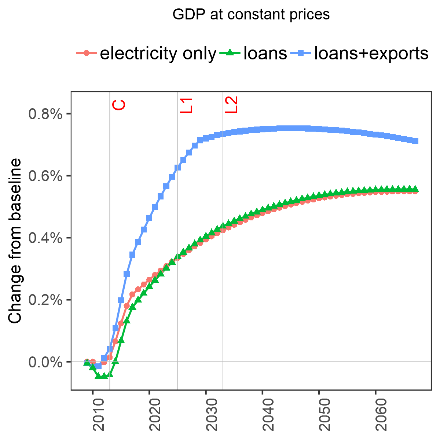 | 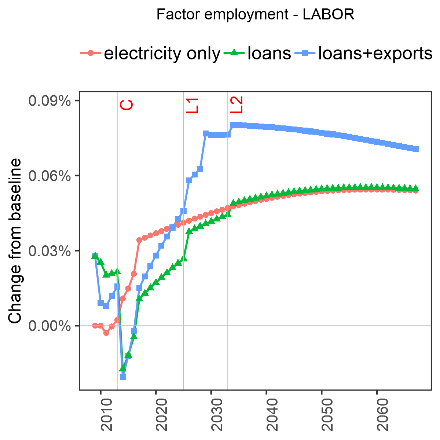 |
| c) General investment | d) Exchange rate |
| 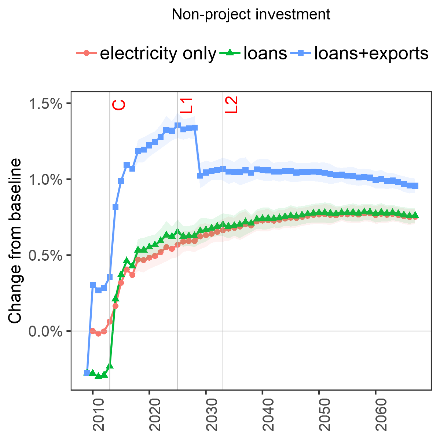 | 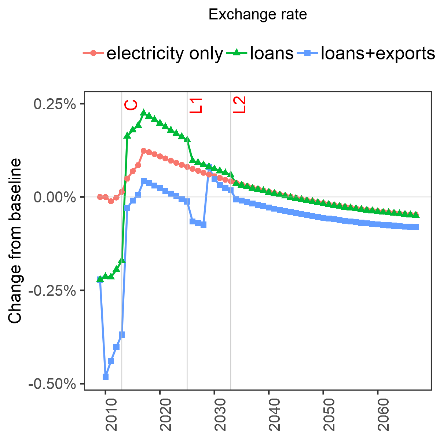 |

Fig. S2 Evolution of macro-economic metrics (GDP, employment, general investment and exchange rate) when considering a deterministic constant hydropower output after the reservoir filling in 2016. This output value is set at the mean annual hydropower generation obtained across the 50 plausible streamflow conditions.

| a) Agriculture* | b) Agrifood | c) Industry |
| --- | --- | --- |
| 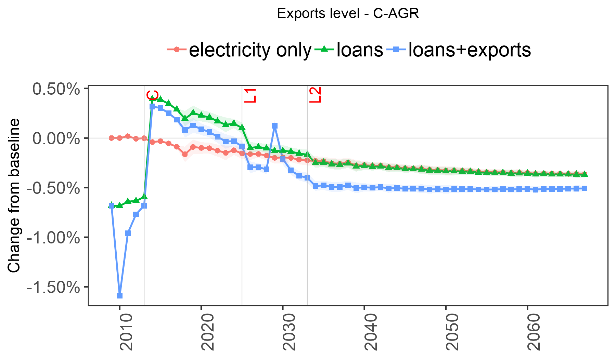 | 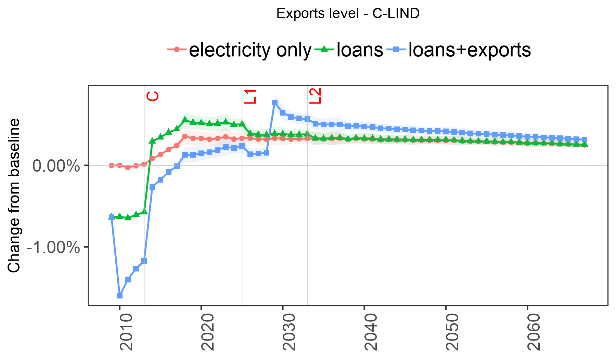 | 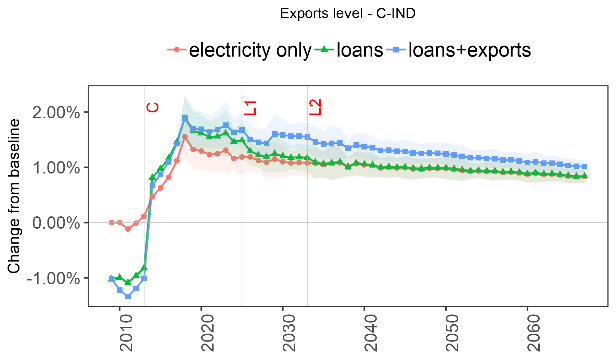 |
| d Electricity | e) Other energy | f) Other services |
| 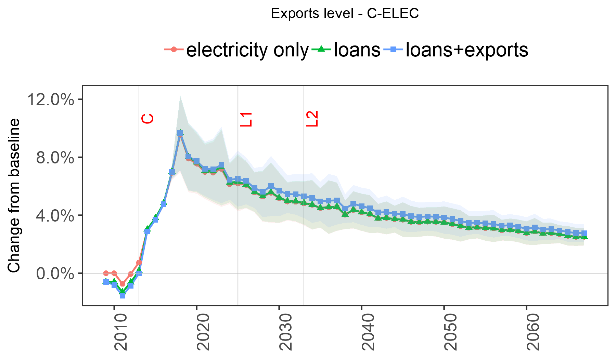 | 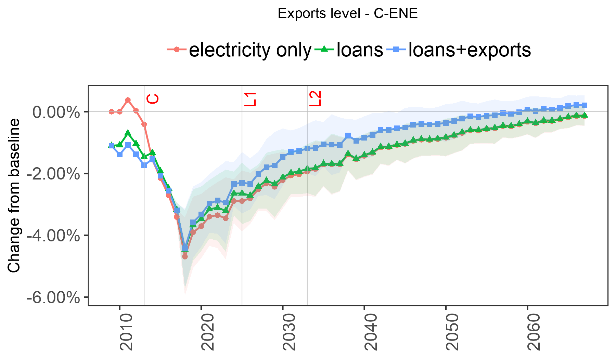 | 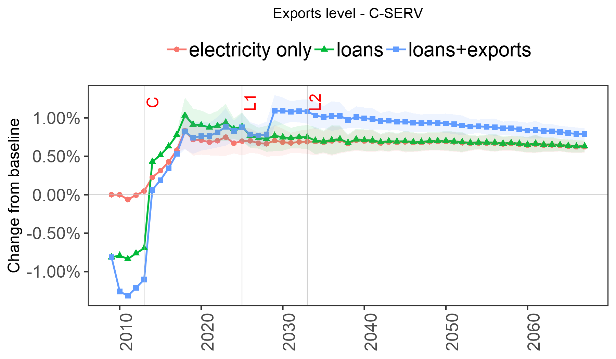 |

Fig. S3 Impacts of financing, construction, and operation of the Bui Dam on the Ghanaian commodity exports. The points represent the mean and the ribbons indicate the standard deviation of the results due to hydrological uncertainty. Note: C = construction completion; L1 = maturity of first loan; L2 = maturity of second loans. *Agricultural exports reflect volumes outside those included in the resource-based loan.

| 1. Cumulated GDP | |
| --- | --- |
| Without demand system recalibration | With demand system recalibration |
| 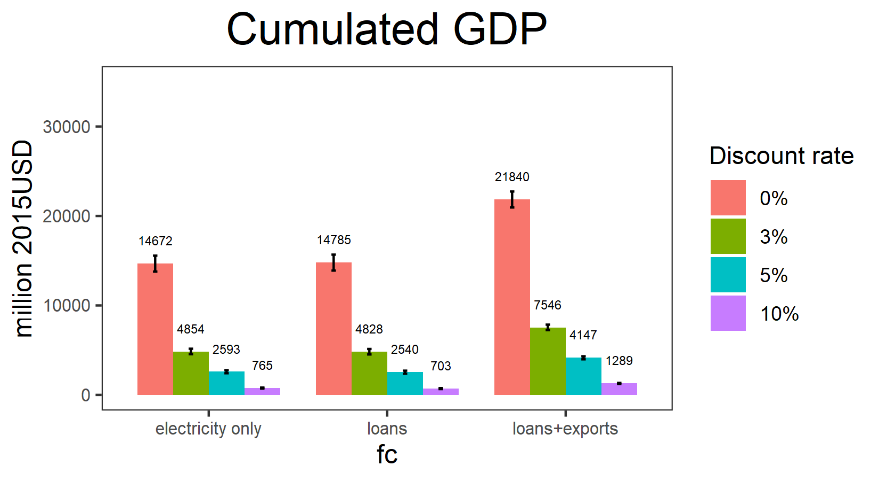 | 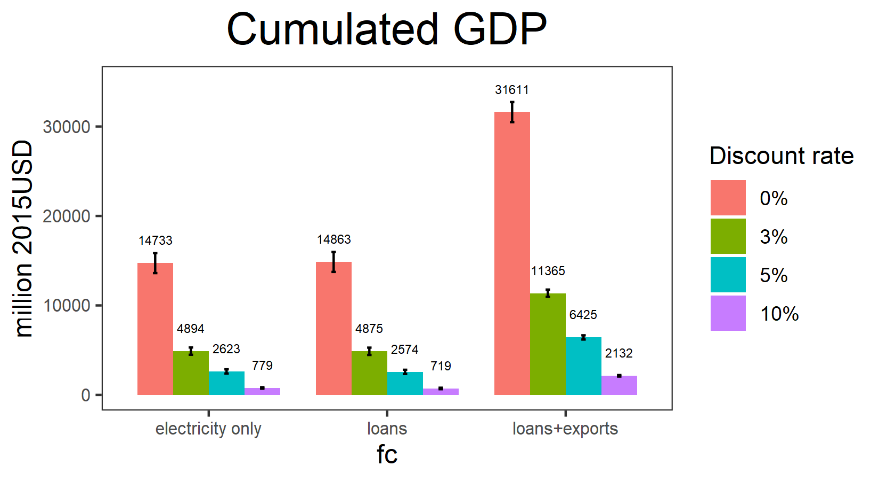 |
| 1. Cumulated household welfare gains | |
| Without demand system recalibration | With demand system recalibration |
| 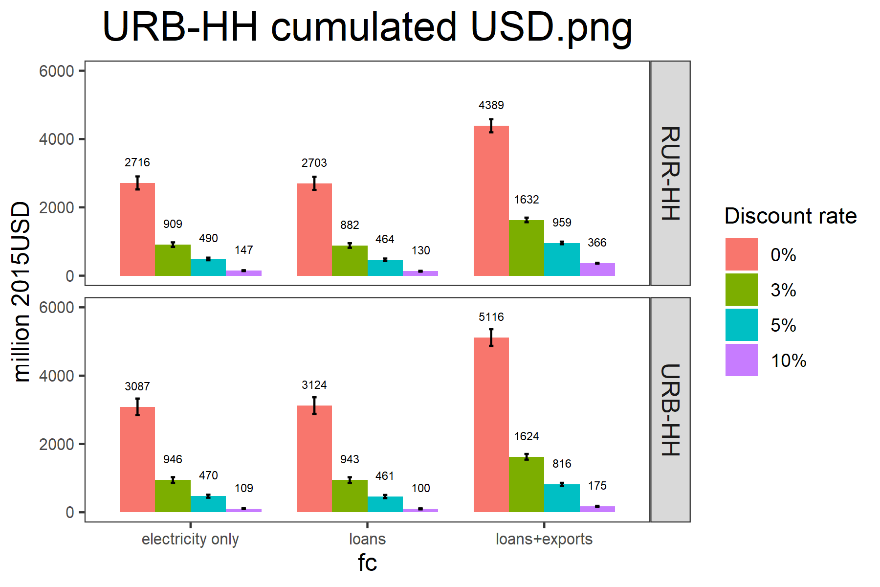 | 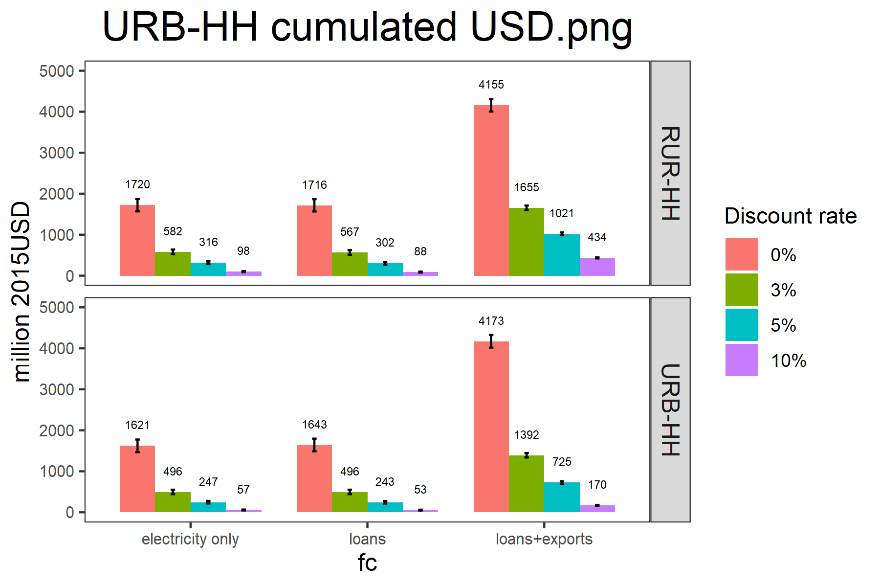 |

Fig. S4 Sensitivity analysis on discount rates and demand system recalibration applied to (a) GDP and (b) household welfare cumulated during the 2009-2065 period. The demand system recalibration implies the recalculation of the subsistence consumption parameters of the LES demand system after each simulation time step.
